# Accurate Recovery of Ribosome Positions Reveals Slow Translation of Wobble-Pairing Codons in Yeast

**DOI:** 10.1089/cmb.2016.0147

**Published:** 2017-06-01

**Authors:** Hao Wang, Joel McManus, Carl Kingsford

**Affiliations:** ^1^ Computational Biology Department, School of Computer Science, Carnegie Mellon University, Pittsburgh, Pennsylvania.; ^2^ Department of Biological Sciences, Carnegie Mellon University, Pittsburgh, Pennsylvania.

**Keywords:** A-site recovery, ribosome profiling, translation rate

## Abstract

**Ribosome profiling quantitatively captures ribosome locations during translation. The resulting profiles of ribosome locations are widely used to study translational speed. However, an accurate estimation of the ribosome location depends on identifying the A-site from ribosome profiling reads, a problem that was previously unsolved. Here, we propose a novel method to estimate the ribosome A-site positions from high-coverage ribosome profiling reads. Our model allows more reads to be used, accurately explains the 3-nt periodicity of ribosome profiling reads from various lengths, and recovers consistent ribosome positions across different lengths. Our recovered ribosome positions are correctly highly skewed toward a single frame within a codon. They retain subcodon resolution and enable detection of off-frame translational events, such as frameshifts. Our method improves the correlation with other estimates of codon decoding time. Furthermore, the refined profiles show that yeast wobble-pairing codons are translated slower than their synonymous Watson–Crick-pairing codons. These results provide evidence that protein synthetic rate can be tuned by codon usage bias.**

## 1. Background

### 1.1. Identifying the active site from ribo-seq reads is a necessary first step

Ribosome profiling is an important sequencing technique that enables various genome-wide translational studies, including on translational response to stress (Ingolia et al., 2009; Gerashchenko et al., 2012; Vaidyanathan et al., 2014), protein synthesis rate (Li et al., 2014), alternative translation initiation (Lee et al., 2012; Gao et al., 2015), translation evolution (Artieri and Fraser, 2014b; McManus et al., 2014), cell development (Brar et al., 2012; Stadler et al., 2012), and the role of specific translation regulation factors (Guo et al., 2010; Guydosh and Green, 2014; Woolstenhulme et al., 2015). The experiment extracts mRNA fragments protected by bound ribosomes (also called ribosome footprints) from RNase I digestion (Ingolia et al., 2009). The technique is analogous to taking snapshots of ribosome locations during translation. Therefore, the ribosome footprint counts at codon locations should be related to the elongation time (Ingolia et al., 2011; Ingolia, 2014). The vector of footprint counts at codon locations of an mRNA is called a ribosome profile, and each individual count is called a ribosome pileup. To date, ribosome profiles are generally used to qualitatively visualize ribosome pauses (e.g., Ingolia et al., 2011; Guydosh and Green, 2014), translation initiation, and translation termination (e.g., Dunn et al., 2013; Albert et al., 2014). Yet, attempts to quantify translation speed, even from the same experiment, often result in controversial conclusions on the determinants of translation rate (Artieri and Fraser, 2014a).

One of the challenges in translation speed quantification is accurate measurement of ribosome decoding locations. Currently, there is no method to extract the precise ribosome decoding locations when the snapshots are taken (Artieri and Fraser, 2014a; Martens et al., 2015). The ribosome P-site or A-site is usually considered the active decoding site (Ingolia et al., 2011; Stadler and Fire, 2011; Michel et al., 2012; Artieri and Fraser, 2014a; Gardin et al., 2014; Lareau et al., 2014; Pop et al., 2014; Martens et al., 2015; Woolstenhulme et al., 2015). This is because the A-site is where the aminoacyl-tRNA enters the ribosome, and the P-site is the position of peptide bond formation. Only the location of either the P-site or the A-site needs to be estimated from the experiment data, and the other one can be inferred.

### 1.2. Imperfect digestions complicate the view of ribosome pileups

Ideally, we expect the read length of ribo-seq data to be identical. For example, the typical ribosome footprint size for yeast is about 28 nt (Ingolia et al., 2009), so we would expect most of ribo-seq in yeast experiments to be 28 bases long. In addition, since ribosomes move in units of codons (3 nt), we expect the ribosome pileups to have a 3-nt periodicity, with most of the reads concentrated on a single base (an in-frame location).

However, ribosome footprint reads do not always share a typical length. For example, in a recently published yeast ribosome profiling data with deep coverage (GSM1335348; Albert et al., 2014), fewer than 60% of uniquely mapped reads have length 28. Furthermore, ribosome footprint reads are not always highly concentrated on a single reading frame. This can be observed from the meta-profiles for the experiment above (Supplementary Fig. S1). A meta-profile is a vector of summed read counts for each location from all transcripts. In this study, reads are grouped by their lengths to examine frame distributions: for each profile, reads are divided into three frames corresponding to the first, second, and third nucleotide of codons, and read length and distribution of these frames are noted above each profile. We observe that although 96% of the reads with length 28 are skewed toward one frame, the highest frame portion for reads with a length not equal to 28 varies from 60% to 80%. While it is possible for reads from the other two frames to be caused by frameshifts, frameshifts are generally rare and thus cannot explain all of the off-frame reads.

In fact, reads with various lengths show unique shapes of 3-nt periodicity. That is, for each of the three bases within a codon, the order of the read abundance often persists across codon locations. For example, if the first frame is the most abundant frame for the first codon, it is also likely to be the most abundant frame for subsequent codons. It is more plausible that the various lengths from ribo-seq reads and the complicated read pileup patterns are caused by imperfect digestions of RNAse I during the experimental procedure. As a result, the read start positions are “redistributed” to off-frame locations. In other words, it is likely that the A-site locations for the majority of the reads are indeed highly concentrated on a single reading frame, but imperfect digestions alter the read start locations, making the observed read pileups to be inconsistent with the actual A-site locations. To sum up, the observations from meta-profiles show that imperfect digestions are very common in ribo-seq data and complicate the view of ribosome pileups.

### 1.3. Existing A-site assignment heuristics cannot explain the complicated read digestion patterns

In past analyses, the A-site location estimation is usually based on simple heuristics. One widely used strategy is that the A-site is simply placed at 15 bases away from the $5 \prime$ end of the footprint read (Ingolia et al., 2009; Stadler and Fire, 2011; Michel et al., 2012; Sabi and Tuller, 2015). This is shown to be accurate for the typical ribosome footprint size (Ingolia et al., 2009). However, as illustrated above, the read length from ribosome profiling experiments can span a wide range (Guydosh and Green, 2014; Lareau et al., 2014; Martens et al., 2015; Woolstenhulme et al., 2015), with as little as $40 \%$ being 28-nt reads (McManus et al., 2014). The A-site position for 28-nt reads might not be suitable for other read lengths.

Since a read length that is not equal to the typical footprint size is mainly caused by incomplete RNase digestion during the experimental procedure (Ingolia, 2014), an alternative strategy is to use a constant A-site offset for a given read length (Ingolia et al., 2011; Dana and Tuller, 2014a; Lareau et al., 2014). This assumes that the digested portion is always the same for all reads with the same length. Such a strategy also implies a 3-nt periodic ribosome position pileup with a highly skewed frame distribution. However, as is observed above, such a frame distribution is not always presented in read pileups for all read lengths. Thus, a large fraction of ribosome footprints have under- or over-digestion (length $\ne  28$), and the simple offset heuristic is insufficient to explain the observed complex frame distribution pattern caused by various nuclease digestion possibilities.

In short, ribosome profiling is a powerful technique to study genome-wide translation mechanisms, but ribosome profiling data are inherently noisy due to complicated experiment pipelines. Specifically, imperfect RNase digestions distort true ribosome profiles and might bury biologically meaningful insights. Such complicated nonuniversal digestions vary between replicates and laboratories and cannot be well captured by existing simple heuristics of A-site assignments.

## 2. Contributions

We introduce a new model and computational method to recover the A-site positions from ribosome profiling data. Our method does not make the incorrect assumption that all reads with the same size are digested to the same extent. Instead, we systematically remove the distortion caused by imperfect digestions and retrieve true ribosome positions. Our procedure results in better A-site position estimation, which enables comparisons of ribosome profiling data from different replicates, conditions, and laboratories, and will hopefully lead to a better understanding of translation speed and regulation.

Observing that read pileups for each read length have a unique start for the 3-nt periodicity, we assume that there is a predominant digestion pattern for each read length. However, individual reads can be over-digested or under-digested to a certain amount, centered around this major digestion pattern. Such an imperfect digestion causes the ribosome A-site to be a variable distance away from the read start. We also assume that there is an unknown underlying true A-site profile consistent across all read lengths. We define this true A-site profile as the ribosome position signal. Such a signal at a particular location is blurred to its surrounding neighborhood due to imperfect RNase digestions. We therefore model the observed read pileups as a blurring of the unknown ground truth positions. We then recover the ground truth positions by combining read pileups from different lengths and allowing the reads to be reallocated with a nonuniversal A-site offset (deblur).

Compared to previous work, our procedure does not assume any specific prior distribution of RNase digestion patterns nor do we assume the imperfect digestion is limited to a 3-nt window (Dana and Tuller, 2014a; Zupanic et al., 2014). Rather, we learn the probabilities of the digestion for each read length from the observed data, enabling a more flexible model to explain the ribosome read pileups. Also, unlike heuristics that discard the off-frame reads (Stadler and Fire, 2011) or take the sum of reads in all three frames (Gardin et al., 2014; Pop et al., 2014), we do not assume that all ribosome reads are from a single reading frame nor do we need to distinguish reads from different frames. Instead, we redistribute reads to their nearby loci, naturally causing the ribosome pileups to be concentrated toward a single frame within a codon. Our approach therefore preserves the subcodon resolution in the estimated A-site positions. We show that on a synthetic frameshift test set, our method retains the frame preferences and strengthens the frame skewness in the estimated A-site profiles.

We showcase our method by estimating codon decoding time (CDT) (Dana and Tuller, 2014a) in yeast ribosome profiling data (Albert et al., 2014). Although abundant tRNAs are expected to speed up codon decoding, the naive global offset heuristic only recovers a weak negative correlation between the tRNA abundance estimates and the CDT. This correlation improves after using our deblurred profiles. Also, for codons decoded by the same tRNA, our estimated CDT shows that the less stable wobble-pairing codons generally translate more slowly than their synonymous codons with Watson–Crick pairing. We find that the difference in decoding time between Watson–Crick-paired codons and wobble-paired codons is generally larger than the difference between two wobble-paired codons. Such phenomena were previously only observed in metazoans (Stadler and Fire, 2011). This observation is consistent with the expectation that wobble pairing is likely to be delayed by the higher probability of tRNA rejection (Tarrant and von der Haar, 2014). Our result therefore provides evidence for the first time in yeast to support such a mechanism. Together, our analysis gives further evidence that frequent codons translate faster than rare codons, and that both tRNA abundance and wobble pairing play roles in elongation speed.

## 3. Methods

### 3.1. Algorithm overview

**FIG. 1.**
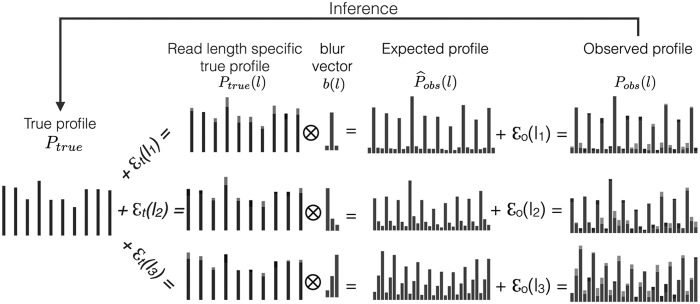
Model of the observed ribosome profiling read pileups. The observed read pileups ${P_{obs}} ( l )$ for read length *l* are modeled as a convolution effect between a blur vector $b ( l )$ and a clear ribosome position signal ${P_{true}} ( l )$. The blur vector diffuses a signal to its nearby locations. The clear signal is somewhat consistent across all read lengths, and can be captured by a consensus clear signal ${P_{true}}$. An additive slack variable ${ \varepsilon _t}$ is used to match ${P_{true}} ( l )$ with ${P_{true}}$, and an additive error ${ \varepsilon _o}$ is used to match the modeled pileups with the observed pileups. Our goal is to extract the consensus clear ribosome positions ${P_{true}}$ from the observed ribosome pileups for all read lengths (${P_{obs}} ( l )$). We call such a clear profile extraction process *Deblur*.

**FIG. 2.**
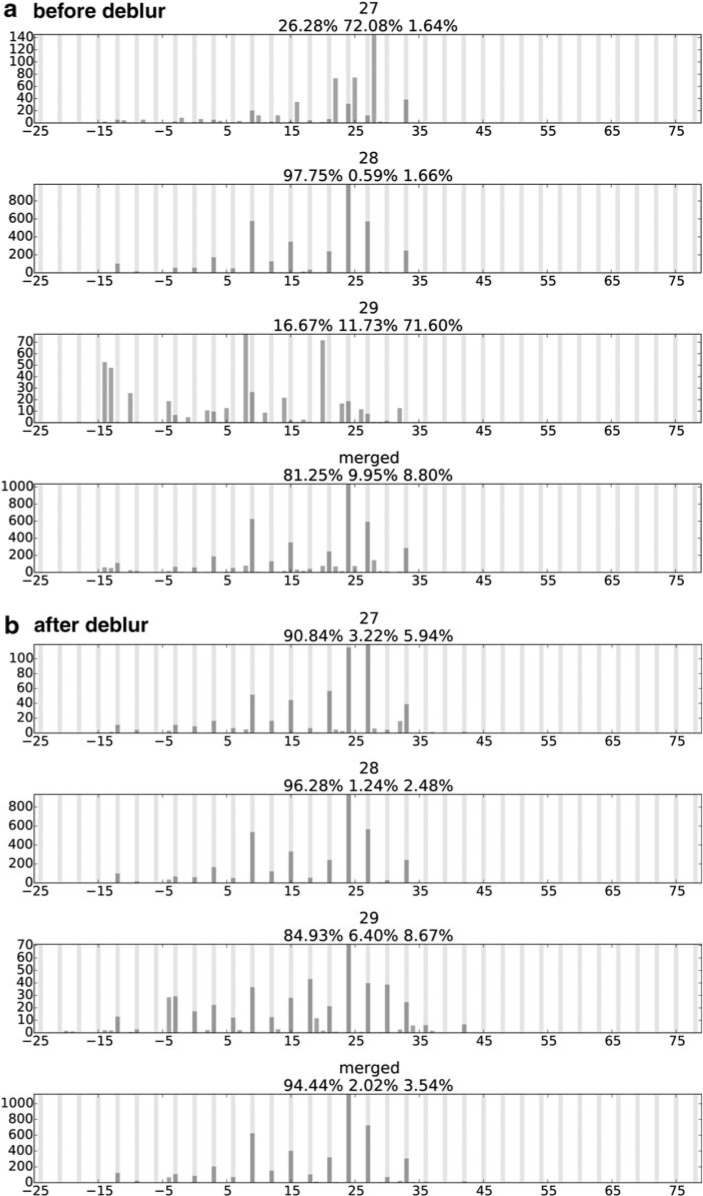
One example of the ribosome profiles for different read lengths before and after debluring on transcript YOR302W. Read length and frame distribution are noted above each profile. In-frame (frame 0) loci are marked with light vertical lines, and read pileups are marked with dark vertical bars. The clear position signal is assumed to be consistent across read lengths, but slight shifts and deviations are allowed.

For a given transcript and each read length *l*, let ${P_{obs}} ( l )$ be the observed ribosome distribution from ribosome profiling reads. We model ${P_{obs}} ( l )$ as the result of a blurring effect on an unknown, length-specific clear ribosome position signal ${P_{true}} ( l )$. We assume such a position signal is consistent across all read lengths and is deviated from an unknown consensus position signal ${P_{true}}$ (Fig. 1). We aim to recover the clear position signal from the observed blurred version of the read positions across all read lengths. The length-specific clear signals ${P_{true}} ( l )$ should be consistent with each other, and our modeled positions ${ \widehat P_{obs}} ( l )$ should agree well with the observed read positions ${P_{obs}} ( l )$. We formulate this task as a total least square optimization problem, where the difference between ${P_{true}}$ and ${P_{true}} ( l )$ and the difference between ${P_{obs}} ( l )$ and ${ \widehat P_{obs}} ( l )$ are simultaneously minimized. We develop an EM-like (expectation maximization) procedure to optimize the objective and to extract the hidden clear position signal ${P_{true}}$ concurrently. One example of our deblur result is shown in Figure 2.

### 3.2. Modeling observed profiles as blurred ribosome position signals

We model the observed ribosome read distribution ${P_{obs}} ( l )$ for read length *l* as a convolution between an unknown clear ribosome position distribution ${P_{true}} ( l )$ and an unknown blur probability vector $b ( l )$: ${ \widehat P_{obs}} ( l ) = b ( l ) *{P_{true}} ( l )$, where $*$ is the convolution operator. The blur vector diffuses the position signal to its neighbor areas. This means, for location *i* on a transcript, the estimated observed ribosome abundance is a linear combination of the nearby true signals:

\begin{align*}
{ \widehat P_{obs}} ( l ) [ i ] = \mathop \sum \limits_{j = - w}^w b ( l ) [ j ] \times {P_{true}} ( l ) [ i - j ] \;,
\end{align*}

where *w* is the width of the blurring effect. The notation $x [ i ]$ indicates the *i*th element of vector *x*.

We require ${P_{true}} ( l )$ to be as consistent as possible across all read lengths. Specifically

\begin{align*}
{P_{true}} ( l ) [ i ] = {P_{true}} [ i - {k_l} ] - { \varepsilon _t} ( l ) [ i - {k_l} ],
\end{align*}

where ${P_{true}}$ is the consensus position signal consistent across all read lengths, ${ \varepsilon _t} ( l )$ is the deviation of ${P_{true}} ( l )$ from ${P_{true}}$ due to length-specific digestion preferences, and *k_l_* is a shift to align profiles with different lengths.

Profiles with different lengths can be aligned by observing that the start of the 3-nt periodicity is read-length specific. We observe from the meta-profiles that the 3-nt periodicity for reads with length *l* starts at $- l + 16$ (Supplementary Fig. S1, darker lines are where 3-nt periodicities start). Therefore, the amount of shift between profile of length *l_1_* and profile of length *l_2_* is $- {l_1} + 16 - ( - {l_2} + 16 ) = {l_2} - {l_1}$. In our model, to align profiles with different lengths, ${P_{true}} ( 28 )$ is used as the anchor, therefore ${P_{true}} ( l )$ can be aligned to ${P_{true}} ( 28 )$ by shifting ${k_l} = l - 28$ to the right. We denote by $P_{true}^{{k_l}}$ and $\varepsilon _t^{{k_l}} ( l )$ the shifted version of the original vectors.

The starts of the 3-nt periodicity also indicate the locations of the majority of the ribosome read $5 \prime$ boundaries when ribosomes start translating. They thus give the most probable A-site offsets for different read lengths. Although these offsets themselves cannot entirely capture the various distances between the A-site and the read boundaries, they serve as a good starting point for explaining the major digestion pattern of a given read length.

Putting everything together, the observed read locations ${P_{obs}} ( l )$ of length *l* are assumed to be generated from the hidden ${P_{true}}$ signal as follows:

\begin{align*}
{P_{obs}} ( l ) = \overbrace { \underbrace { ( P_{true}^{{k_l}} - \varepsilon _t^{{k_l}} ( l ) ) }_{{P_{true}} ( l ) }*b ( l ) }^{{{ \widehat P}_{obs}} ( l ) } + { \varepsilon _o} ( l ),
\end{align*}

where ${ \varepsilon _o} ( l )$ is the deviation of the modeled profile ${ \widehat P_{obs}} ( l )$ from the observed profile ${P_{obs}} ( l )$. In short, the hidden consensus ${P_{true}}$ is shifted with an additive difference ${ \varepsilon _t} ( l )$, convolved with a blur vector $b ( l )$ to get the modeled profile ${ \widehat P_{obs}} ( l )$, and the difference between the observed profile ${P_{obs}} ( l )$ and the modeled profile is then measured with an additive error ${ \varepsilon _o} ( l )$. The parameters *k_l_*, ${ \varepsilon _t} ( l )$, ${ \varepsilon _o} ( l )$, $b ( l )$, must be optimized to find the hidden ${P_{true}}$. We explained above the rationale of choosing *k_l_*, and we describe how other parameters are optimized in the following sections.

### 3.3. Deblurring ribosome profiles—a least square optimization

Our goal is to use the blurred observed profiles ${P_{obs}} ( l )$ to deconvolve the clear ribosome position signal ${P_{true}}$ of a transcript. Such clear signals should be consistent across all read lengths and should be a good estimate of the observed ribosome distribution after applying the blurring effect. The consensus clear position signal ${P_{true}}$ and the deviation between the consensus and the length-specific ribosome signal (${ \varepsilon _t} ( l )$) are adjusted to minimize two terms: the difference between the observed profile and the modeled profile and the difference between the consensus and the length-specific ribosome signal. Specifically

\begin{align*}
\min \limits_{{{P_{true}}}, \ \varepsilon _t ( l ) } \mathop \sum
\limits_l \alpha ( l ) \left[  \parallel  P_{obs} ( l ) -
{\widehat P_{obs}} ( l ) \parallel _2^2 + \parallel P_{true}^{k_l}
- P_{true} ( l ) \parallel _2^2 \right] , \tag{1}
\end{align*}

where $\alpha ( l )$ is the total read count with length *l* for the tested transcript. Intuitively, if some read length is more abundant, the true position signal recovered from that read length should be weighted more.

Using ${P_{true}} ( l ) = P_{true}^{{k_l}} - \varepsilon _t^{{k_l}} ( l )$ and ${ \widehat P_{obs}} ( l ) = b ( l ) * ( P_{true}^{{k_l}} - \varepsilon _t^{{k_l}} ( l ) )$, we rewrite (1) to be

\begin{align*}
\mathop {\min} \limits_{{{P_{true}}} ,{\varepsilon _t} ( l ) }
\mathop \sum \limits_l \alpha ( l ) \left[ { \parallel {P_{obs}} (
l ) - b ( l ) * ({ P_{true}^{{k_l}} - \varepsilon _t^{{k_l}} ( l
)} )  \parallel _2^2 + \parallel {{\varepsilon _t^{{k_l}}} ( l )}
\parallel _2^2} \right] . \tag{2}
\end{align*}

If the blur vectors $b ( l )$ are known, we can use an EM-like framework to find the least square solution:

**M-step:** We fix ${P_{true}}$ and adjust ${ \varepsilon _t} ( l )$ to optimize the total least square problem in (2), where ${ \varepsilon _t}$ for each *l* can be optimized separately:

\begin{align*}
\mathop { \min } \limits_{{ \varepsilon _t} ( l ) }  \parallel  b
( l ) * {\varepsilon _t^{{k_l}} ( l )} - ( b ( l )
*P_{true}^{{k_l}} - {P_{obs}} ( l ) )  \parallel _2^2 + \parallel
{\varepsilon _t^{{k_l}} ( l )} \parallel _2^2 , \tag{3}
\end{align*}

where bold indicates the variables we are optimizing. The optimal ${ \varepsilon _t} ( l )$ is found via a least square solver with Ridge regression (Fong and Saunders, 2011) (${ \rm{damp}} = 1$).

**E-step:** We fix ${ \widehat P_{obs}} ( l )$ and ${P_{true}} ( l )$ as estimated from the M-step and adjust the consensus ${P_{true}}$ to minimize the objective in (1). The expected ${P_{true}}$ is therefore the weighted average of all ${P_{true}} ( l )$. After the M-step, the new estimation of ${P_{true}} ( l )$ is $P_{true}^{{k_l}} - \varepsilon _t^{{k_l}} ( l )$, so the weighted average of ${P_{true}} ( l )$ is as follows:

\begin{align*}
P^\prime _ {true} = \mathop \sum \limits_l { \frac {\alpha ( l ) (
P_ {true} ^ {{ k_l } } - \varepsilon _t^ { { k_l } } ( l ) ) } {
\sum \nolimits_ { l^\prime } { \alpha ( l^\prime ) } } } = { P_ {
true } } - \mathop \sum \limits_l { \frac { \alpha ( l )
\varepsilon _t^ { { k_l } } ( l ) }  { \sum \nolimits_ { l^\prime
} { \alpha ( l^\prime ) } } } . \tag { 4 }
\end{align*}

We set all negative entries of ${P^\prime _{true}}$ to be zero and renormalize ${P^\prime _{true}}$ so that it sums to 1. This constrains ${P_{true}}$ to remain valid and in practice appears to have a minor effect on the shape of ${P_{true}}$.

We repeat the EM-like procedure until the change of the objective in (2) compared to the objective value from the previous step is smaller than 0.01.

We initially set ${P_{true}}$ to be the in-frame values of the observed read pileups with length 28:

\begin{align*}
{P_{init}} [ i ] = \left\{  { \begin{matrix} {{P_{obs}} ( 28 ) [ i ] } & { \quad { \rm{if }}\ i \ { \rm{ is \ a \ multiple \ of \ 3}},} \\   0 & {{   \rm{otherwise.}}} \\ \end{matrix} } \right. \tag{5}
\end{align*}

${P_{obs}} ( 28 )$ is used as the initial consensus because 28 is the typical ribosome footprint size for yeast. For such size, the real physical ribosome footprint boundaries should be most likely to overlap with the read ends. This is because an imperfect digestion for reads with length 28 has to be caused by a simultaneous over-digestion from one end and an under-digestion from the other end, which is likely to be relatively rare. Therefore, the observed read pileups with read length 28 should be the most clear and the closest to the ground truth position signal. Indeed, these profiles show the strongest frame skewness and the most visible 3-nt periodicity (Supplementary Fig. S1).

### 3.4. Estimating blur vectors from meta-profiles

The deblur process depends on a known set of blur vectors ($b ( l )$)—a crucial element to model the imperfect digestion in ribosome reads. These vectors describe the probability of relocating ribosomes to transfer the clear ribosome position signal to the observed read pileups. Since these pileups are read $5^\prime$ end pileups (see the 3rd paragraph), essentially the blur vectors adjust a true footprint boundary to the observed read boundary. They therefore indicate the probability of the amount of under/over-digestion from the $5^\prime$ end and capture the read-length-specific digestion patterns.

These blur vectors can be estimated directly from the ribosome reads via meta-profiles. Meta-profiles are widely used to reveal the positional patterns of ribosome profiles (Ingolia et al., 2009, 2011; Guo et al., 2010; Dana and Tuller, 2012; Shah et al., 2013; Gerashchenko and Gladyshev, 2014; Lareau et al., 2014). They do so by summing read pileups from all transcripts for each position. The blur vectors can be estimated from these meta-profiles because convolution satisfies the distributive property.

To generate the meta-profiles, we group reads by lengths and accumulate the positions of the $5 \prime$ ends relative to the start codon for all transcripts. We then include the first 350 locations away from the start codon in the meta-profiles. We only use transcripts with length $> 350$ to reduce the convolution boundary effect. Also, to avoid the outlier points biasing the shape of the blur vector, we exclude locations in the meta-profiles with the top 1.65% highest read counts. This threshold is chosen by assuming the top 5% of in-frame reads (1/3 of total reads) are outliers.

To estimate the blur vectors, we use an EM-like procedure similar to the earlier deblur optimization. In this procedure, the observed transcript profiles are replaced by the meta-profiles, and the blur vectors are adjustable variables. The procedure is exactly the same as described in the previous section, except that the blur vector is first estimated before the M-step:

\begin{align*}
\mathop { \min } \limits_{b ( l ) }  \parallel  M_{true}^{{k_l}}*b{ \bf{ ( }}l{ \bf{ ) }} - {M_{obs}} ( l )  \parallel _2^2 ,
\end{align*}

where the “*M*” variables are the meta-profiles, and we replace ${P_{true}}$ by ${M_{true}}$ and ${P_{obs}} ( l )$ by ${M_{obs}} ( l )$ in (3)–(5). The blur vector size, which limits the diffusion range of the position signal, is set to 31. This way the signal can be diffused, either to the left or to the right, as far as approximately half the size of a ribosome. A non-negative least square solver (scipy.optimize.nnls) is used to find the best $b ( l )$. All blur vectors with different read lengths are optimized separately.

### 3.5. Estimating the A-site profile

We merge the length-specific true profiles to get an overall ribosome position signal for a given transcript—the A-site profile. It is the weighted sum of all the length-specific true profiles, shifted to the right by 15:

\begin{align*}
{C_{true}} [ i ] = \mathop \sum \limits_l \alpha ( l ) {P_{true}} ( l ) [ i + {k_l} - 15 ].
\end{align*}

The shift is needed since the true profiles are estimated from the reads' $5^\prime$ ends, and ${P_{true}} ( 28 )$ is the anchor to align profiles with different length. We shift by 15 since it is the major A-site offset of reads with length 28, and it is the A-site offset under perfect digestion.

### 3.6. CDT estimation

To investigate the influence of tRNA abundance and wobble pairing on translation speed, we estimate CDT using the procedure in Dana and Tuller (2014a). The in-frame (frame-0) deblurred read counts are used as the input ribosome count for each codon position. Such counts are normalized by the average ribosome count for each transcript, as is done in Lareau et al. (2014) and Woolstenhulme et al. (2015). Following Dana and Tuller (2014a), these normalized counts are grouped by codon types to form codon count distributions, with the exclusion of the first and last 20 codon positions of each transcript and positions with ribosome counts less than 1. Each codon distribution is fit with a log normal distribution. The skewness of the log normal distribution is used as an estimate of the CDT, as it has been shown to be informative for estimating the elongation speed from ribosome profiling data among various species (Dana and Tuller, 2014a).

### 3.7. Read alignment and data preprocessing

We test the deblur method on ribosome profiling data from *Saccharomyces cerevisiae*, where ambiguous mapping is not ubiquitous. We use ribosome reads from a yeast study, where the data are of high quality and of high sequencing depth (GSM1335348) (Albert et al., 2014).

Reads were first aligned to the yeast noncoding RNA reference, which includes rRNA, tRNA, snoRNA, and so on, to remove noncoding contaminants. The remaining reads are then mapped to the yeast transcriptome. The yeast noncoding RNA reference and the transcriptome reference are downloaded from the Saccharomyces Genome Database (Engel and Cherry, 2013). Alignments are performed with STAR (Dobin et al., 2013) with parameters—clip3pAdapterSeq CTGTAGGCACCATCAAT—outFilterMismatchNmax 1, which automatically “softclips” the unaligned adapter sequences and any unaligned bases at the $5^\prime$ end of the reads, allowing at the most 1 mismatch. Only uniquely mapped reads, about $83 \%$ of the noncontaminated reads, are used to generate the observed profiles ${P_{obs}} ( l )$.

The observed profile of a given length is included for a transcript in the deblur process if more than 50% of the in-frame loci have nonzero ribosome counts. In this study, we define “in-frame” as the frame with the highest total read count. Only transcripts with at least two observed profiles from different read lengths are tested for deblur.

Such filtering results in 1966 transcripts with high ribosome coverage. This transcript set size agrees with the size of highly expressed transcript set: 2108 transcripts share an estimated expression level $> 100$ transcript per million [expressions are estimated using Salmon (Patro et al., 2015) with the RNA-seq data from the same experiment (GSM1335347; Albert et al., 2014)].

## 4. Results

### 4.1. Ribosome profiles are well explained by the blur model

**FIG. 3.**
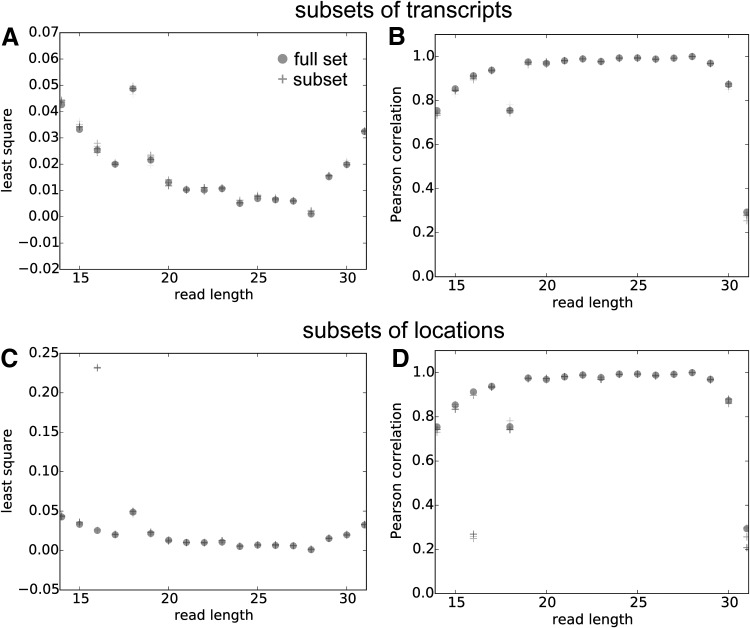
The agreement between the observed meta-profiles and the modeled meta-profiles with blur vectors trained on subsamples of data. **(A, B)** The transcripts are randomly divided into five groups with equal size, and the blur vectors are trained on the subset of transcripts. **(C, D)** The profile locations are randomly divided into five groups with equal sizes, and the blur vectors are trained on the subset of locations. The least square and the Pearson correlation are between the observed meta-profiles (${M_{obs}} ( l )$) and the modeled meta-profiles (${ \widehat M_{obs}} ( l ) = {M_{init}}*b ( l )$) for each read length *l*. Results are qualitatively similar regardless of whether the blur vectors are trained on a subsample of the data. Read length 18 is modeled slightly worse primarily due to low coverage and significant outliers.

We test whether the estimated blur vectors can truthfully characterize the observed read locations on the meta-profiles. In this study, different blur vectors are convolved with the initial guess of the meta-consensus—the in-frame values of ${M_{obs}} ( 28 )$. Our modeled meta-profiles agree well with the observed meta-profiles. The blur process results in a good correlation and a small deviation between the modeled meta-profiles and the observed meta-profiles across all read lengths (Fig. 3).

To test whether a single blur vector is sufficient to model all profiles for a given length, we train the blur vector on subsets of locations and on subsets of transcripts, and we get similar results compared to training the blur vector on the entire set (Fig. 3). This indicates that the blur vectors are transcript and location independent.

**FIG. 4.**
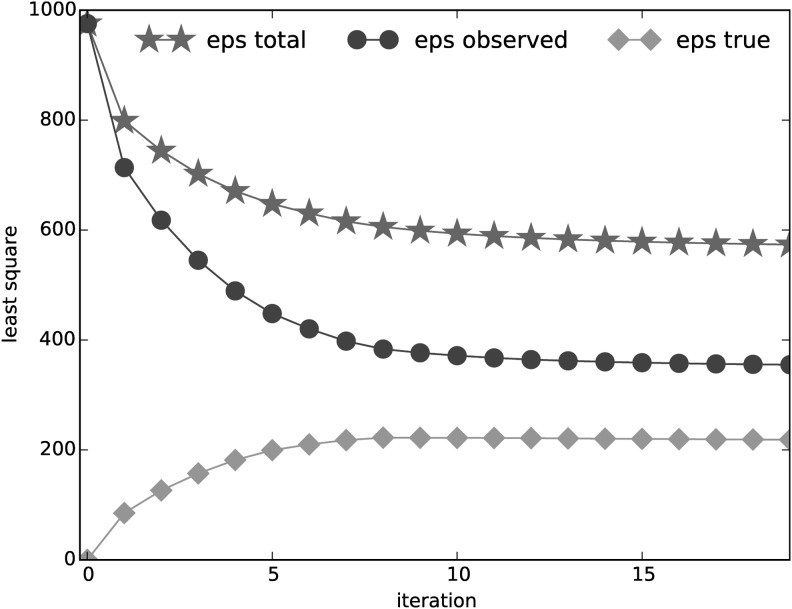
The change of the objective function in Equation (1) for deblurring the meta-profiles. The circle line is the overall inconsistency between the modeled meta-profile (${ \widehat M_{obs}} ( l )$) and the observed meta-profile (${M_{obs}} ( l )$), the diamond line is the overall inconsistency between the consensus clear signal ($M_{true}^{{k_l}}$) and the read-length-specific deblurred signal (${M_{true}} ( l )$), and the star line is the sum of the 2. The overall inconsistency between the modeled meta-profiles and the observed meta-profiles successfully goes down during the optimization, and the observed profiles are better modeled by sacrificing the inconsistencies of clear signals across different lengths.

**FIG. 5.**
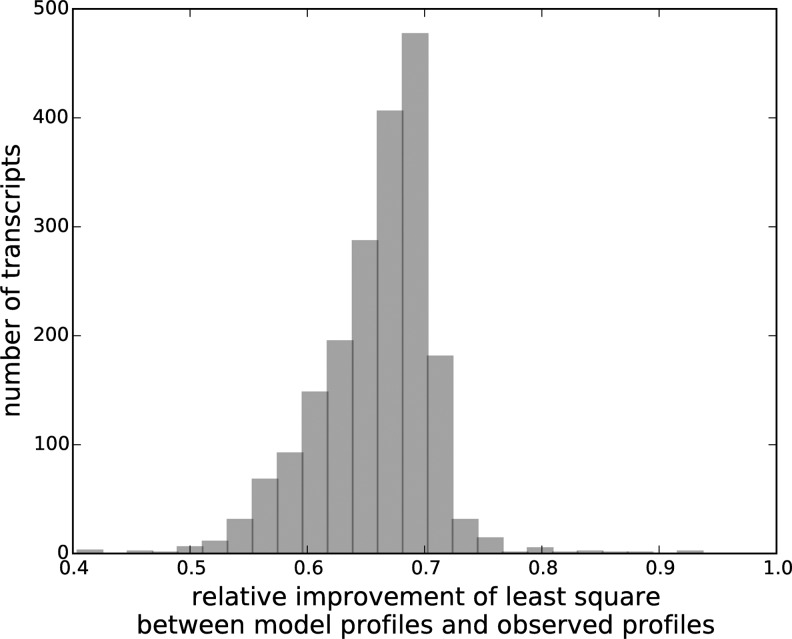
Histogram of the relative improvement of the overall inconsistency between the observed profiles ${P_{obs}}$ and the modeled profiles ${ \widehat P_{obs}}$ after the deblur procedure. The relative improvement is defined as the change of the inconsistency between ${P_{obs}}$ and ${ \widehat P_{obs}}$, over the initial inconsistency between the two.

In addition, allowing the length-specific clear position signals to be slightly deviated from the consensus further improves our model fitting. Instead of enforcing an identical consensus across all read lengths, these deviations result in both better modeled meta-profiles (Fig. 4) and better modeled transcript profiles (Fig. 5): the inconsistencies between the observed profiles and the modeled profiles are reduced by an average of 66% compared to not allowing such deviations.

### 4.2. Consistent read-length-specific profiles

To test how shifting and deblurring affect the consistencies among profiles with different read lengths, we compare read-length-specific profiles with the in-frame values of the observed profiles of length 28 (${P_{init}}$, Eq. (5)). We choose ${P_{init}}$ for comparison because it is the original data in which we have the most confidence. We use the Pearson correlation coefficient as a measurement of the consistency between the read-length-specific profile and ${P_{init}}$.

**FIG. 6.**
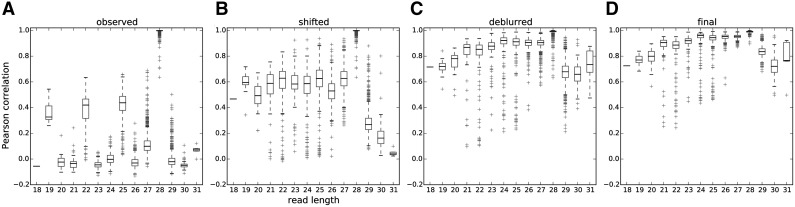
Effect of shifting and deblurring on profile consistencies across different read lengths. ${P_{obs}} ( l )$ is the observed profile of length *l*, ${P_{init}}$ is the in-frame value of ${P_{obs}} ( 28 )$, which is also the initial guess of the true profile, *k_l_* is the shift applied to a profile, ${P_{true}} ( l )$ is the length-specific deblurred profile, and ${P_{true}}$ is the consensus of ${P_{true}} ( l )$s. Bar plots of the Pearson correlation are between **(A)** ${P_{obs}} ( l )$ and ${P_{init}}$, **(B)** $P_{obs}^{{k_l}} ( l )$ and ${P_{init}}$, **(C)** $P_{true}^{{k_l}} ( l )$ and ${P_{init}}$, and **(D)** $P_{true}^{{k_l}} ( l )$ and ${P_{true}}$. The improvement of the Pearson correlation between the read-length-specific profiles and the true profiles is the combinational effect of the right amount of shifts and the success of deblurring.

Two factors jointly improve the consistencies of ribosome profiles among different lengths: the deblur process, and allowing a length-specific shift and deviation from the consensus. Initially, none of the raw observed profiles (${P_{obs}} ( l )$) correlates well with the in-frame values of ${P_{obs}} ( 28 )$ (Fig. 6A). However, the correlations are improved if the observed profiles are properly shifted and aligned to ${P_{obs}} ( 28 )$ (Fig. 6B). The correlations can be further increased by applying the deblur process to recover the length-specific clear profiles (${P_{true}} ( l )$) (Fig. 6C). Finally, compared to the initial guess of the consensus (${P_{init}}$), at the end of the deblur process, the final consensus estimation (${P_{true}}$) correlates better with the length-specific clear profiles (Fig. 6D).

Overall, the correlation between ${P_{true}} ( l )$ and ${P_{true}}$ for most lengths is close to 1. Since ${P_{true}}$ is the centroid of ${P_{true}} ( l )$, the good correlation between the two indicates that the deblurred profiles are consistent across different read lengths.

### 4.3. Improved frame skewness

**FIG. 7.**
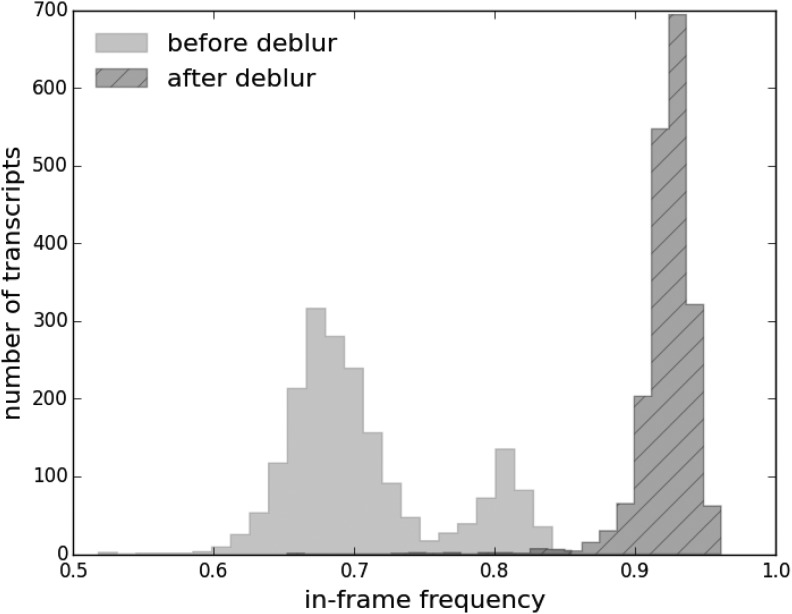
Histograms of in-frame (frame 0) portion of reads before and after deblur. The recovered A-site profiles have a higher in-frame skewness compared to the original profiles.

Our deblur process improves the frame skewness of the recovered A-site profiles, even if it does not explicitly optimize or force frame skewness. The in-frame position is the reading frame where reads are preferentially distributed within a codon. It is usually frame 0 for the A-sites of ribosome footprints with the absence of frameshifts. It is desirable for the recovered ribosome A-site profiles to be highly skewed toward frame 0. This is because ribosomes move in units of codons, so ribosome profiles should have 3-nt periodicity. Also, such profiles should be mainly concentrated on frame 0, since frameshifts are rare. Indeed, after deblur, the in-frame skewness does improve from an average of 71% to 92% (Mann–Whitney *U* test $p < 3 \times {10^{ - 308}}$; Fig. 7). This indicates that the deblur process produces ribosome profiles with less noise. It also enables more reads to be used in downstream analysis. For instance, if only the in-frame reads are used to represent the codon-level ribosome counts, the deblur process will allow on average 20% more reads to be used.

### 4.4. Deblur process produces subcodon resolution profiles

**FIG. 8.**
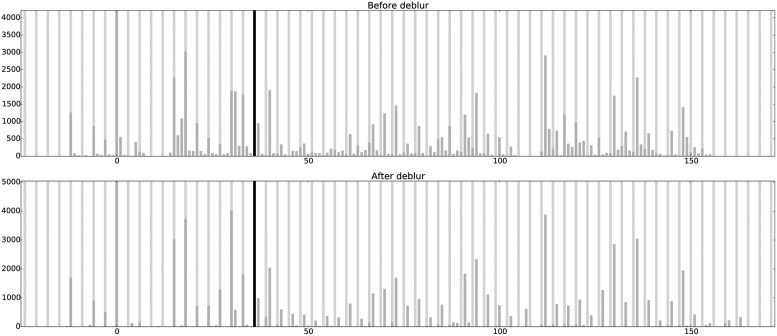
An example of the ribosome profiles with artificially programmed frameshift on transcript YDL061C before and after deblur. Frame-0 loci are marked with light (vertical lines) and the frameshift point is marked with a darker vertical line. The high frame-0 skewness before the frameshift point and the high frame-1 skewness after the frameshift point are well maintained and strengthened by the deblur process.

The deblur procedure does not assume that the recovered A-site profiles are all from a fixed frame, and thus, it keeps the subcodon resolution of the A-site profiles and allows detection of potential programmed frameshifts. To test whether the deblur process can recover profiles with frameshifts, we synthetically generate frameshifts as follows: we first choose a random frame-0 location as the frameshift point in a transcript and then shift all reads with a start location after such point to the right. This is to simulate an insertion in the transcript to induce a frameshift. The recovered A-site profiles should have a high skewness toward frame 0 before the frameshift point, and a high skewness toward frame 1 after the frameshift point. One example of our constructed frameshift events is shown in Figure 8.

**FIG. 9.**
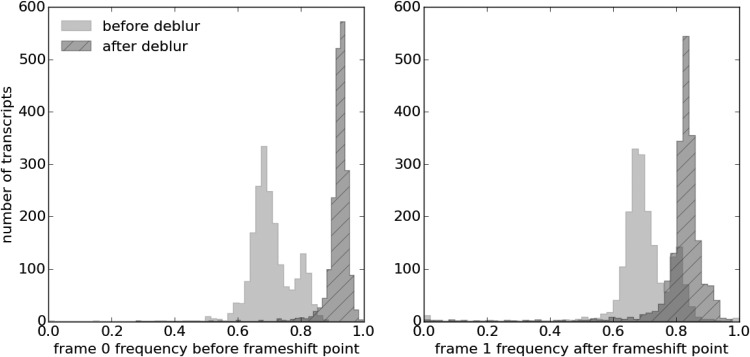
Histograms of the frame-0 portion of the ribosome profiles before the frameshift point and the frame-1 portion of the ribosome profiles after the frameshift point. The deblur process strengthens the frame skewness while keeping the estimated ribosome positions to be in the correct frame.

The deblur process successfully maintained and improved the skewness of frame 0 before the frameshift point and the skewness of frame 1 after the frameshift point (Fig. 9). Therefore, combining profiles with lengths other than 28 during the deblur process results in a recovery of a clear frameshifted A-site profile, regardless of the incorrect initial guess. To sum up, frameshift detection is an important task, but the current frameshift detection method (Michel et al., 2012) suffers from high false-positive rates. Our deblur process recovers ribosome profiles with a clear frame preference, which will promote the development of a better frameshift detection.

### 4.5. Wobble-pairing codons translate slower than Watson–Crick pairing

The tRNA abundance was expected to be negatively correlated with the CDT (Dana and Tuller, 2014a,b; Gardin et al., 2014; Lareau et al., 2014), and such correlation is strengthened using our deblurred profiles. After deblur, $85 \%$ of the codon distributions have a smaller variance, indicating that the deblur process successfully removes noise from the observed read pileups. From these distributions, the estimated CDT is the skewness of a lognormal fit (Dana and Tuller, 2014a) (details in Section 3). Such estimated CDT is compared with the tRNA Adaptation Index (tAI) (dos Reis et al., 2004)—a proxy for the tRNA concentration. The deblur process strengthens the Spearman correlation between the tAI and the estimated CDT from −0.21 ($p = 0.1$) to −0.46 ($p = 1 \times {10^{ - 4}}$). This provides stronger evidence that tRNA abundance play a role in elongation speed. Similarly, the raw frequency of codon usage also negatively correlates with the estimated CDT (Spearman correlation $- 0.5$, $p = 3.7 \times {10^{ - 5}}$), indicating frequent codons are translated faster than rare codons.

Wobble pairing could also affect the elongation speed. Since there are usually fewer tRNA types than codon types, some of the codons that encode the same amino acid must be decoded by the same tRNA. Wobble pairing allows a tRNA to recognize more than one codon. Within these synonymous codons, the determinant of the codon decoding speed is the efficiency of the tRNA recognizing the corresponding codon. According to the Wobble hypothesis (Crick, 1966), the last two bases of the tRNA anticodon form Watson–Crick base pairs and bond strongly to the first two bases of the codon. However, the anticodon's first base can form a wobble pair: the base G can either Watson–Crick pair with C or wobble pair with U; the base I (inosine, edited from A) can also both wobble pair with C and U, but I:U pairing has a less favorable geometry (Stadler and Fire, 2011); the base U can Watson–Crick pair with A and wobble pair with G. It has been hypothesized that wobble-paired codons tend to be translated slower than their synonymous Watson–Crick paired codons, since wobble pairs are more likely to be rejected before peptidyl transfer, causing the tRNA selection cycle to be repeated (Tarrant and von der Haar, 2014).

**FIG. 10.**
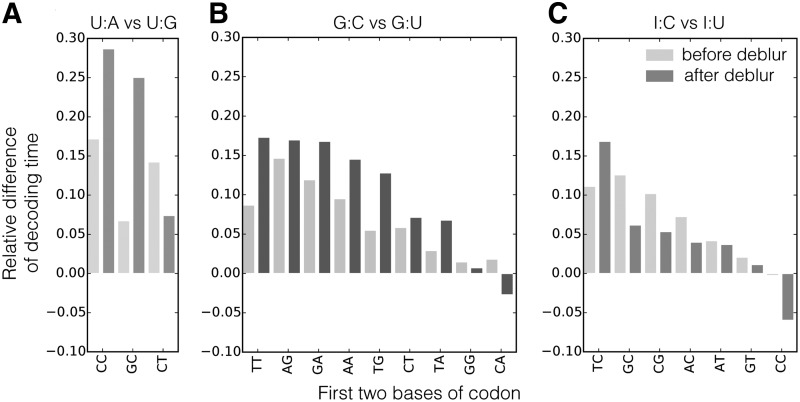
Relative differences of the CDT between pairs of codons that are decoded by the same tRNA. Lighter colors are time differences estimated from original ribosome profiles, and darker colors are time differences estimated from deblurred profiles. The anticodon: codon pairings are **(A)** U:A (Watson–Crick) versus. U:G (wobble), **(B)** G:C (Watson–Crick) versus G:U (wobble), **(C)** I:C (stronger wobble) versus I:U (weaker wobble). The average relative time difference estimated from the deblurred profiles is 0.2 between U:A and U:G, 0.1 between G:U and G:C, and 0.04 between I:U and I:C; the average relative time difference estimated from the original profiles is 0.12 between U:A and U:G, 0.07 between G:U and G:C, and 0.07 between I:U and I:C.

We investigated how wobble pairing influences CDT in yeast. We focus on pairs of codons that are translated by the same tRNA, so that the influence of tRNA concentration on the elongation speed is controlled. In this case, the codon pair shares the first two bases and differs in the third base. We compared the estimated decoding time between the codon pairs and find that the wobble-pairing codons indeed are estimated to often translate slower than the Watson–Crick pairing codons (Fig. 10).

We expect the decoding time difference between two wobble-paired codons to be smaller than the difference between a wobble-pair codon and a Watson–Crick pair codon, if the wobble-paired tRNA is truly more likely to leave the ribosome without successful peptidyl transfer (Tarrant and von der Haar, 2014). For the three codon pairs being compared, we would therefore expect the time difference between I:C and I:U to be smaller than the time difference between G:C and G:U, and between U:A and U:G. To control for the absolute level of the translation time, we use the relative decoding time difference between a synonymous codon pair. It is defined as follows: $\Delta t = ( {t_{{ \rm{wobble}}}} - {t_{{ \rm{Watson}} - { \rm{Crick}}}} ) / {t_{{ \rm{Watson}} - { \rm{Crick}}}}$, where *t_x_* is the estimated decoding time for a codon with either wobble pairing or Watson–Crick pairing.

Using the profiles from the deblur process, the decoding time difference is inline with the above expectation. The decoding time difference between a wobble-paired codon and a Watson–Crick paired codon is indeed visibly larger than the decoding time difference between two wobble-pair codons (Fig. 10). Although such a trend was first seen in metazoans (Stadler and Fire, 2011), it was not observed for most wobble-paired codons in yeast (Artieri and Fraser, 2014a; Gardin et al., 2014; O'Connor et al., 2015). It is also less obvious when CDTs are estimated from the original ribosome profiles (Fig. 10). This indicates that the uncorrected ribosome profiles obscure true ribosome A-site positions. Together, the CDT estimated from the deblurred profiles strengthens the conclusion that wobble pairing slows translation. These results also suggest that wobble pairing can be used as a mechanism to regulate elongation speed.

## 5. Discussion

Estimating ribosome A-site positions from ribosome profiling data is a challenging necessary step in quantifying codon-specific translation speed and ribosome pausing. There are controversial conclusions about whether the tRNA level plays an important role in CDT. Different analysis pipelines performed on different experiments show that the estimated CDT sometimes strongly correlates with the codon usage (Gardin et al., 2014), sometimes weakly correlates with tAI (Lareau et al., 2014), and sometimes does not correlate with the codon optimality (Artieri and Fraser, 2014a) or tRNA level (Pop et al., 2014). Different estimates of CDT alone produce different correlations between the estimated decoding time and the tRNA level among different species (Dana and Tuller, 2014a,b). The fact that there is evidence both for and against the correlation between tRNA levels and CDT indicates that a better CDT analysis pipeline is needed.

We hereby no means try to touch all aspects of the elongation time estimation nor do we try to emphasize or diminish the impact of tRNA level on the translation dynamics. We focus on recovering the A-site positions from the ribosome profiling data, the first step of any quantitative analysis on codon rate or pausing strength. We show via several lines of intrinsic and extrinsic evidence that our deblur method provides better estimates of A-site profiles, leading new insights on translation dynamics. Source code for the deblur method and the analysis can be found at www.cs.cmu.edu/∼ckingsf/software/riboasitedeblur/.

## Supplementary Material

Supplemental data
