# Supplementary figures and images for: Accurate Recovery of Ribosome Positions Reveals Slow Translation of Wobble-Pairing Codons in Yeast
Source: J Comput Biol. 2017 Jun 1;24(6):486–500. doi: 10.1089/cmb.2016.0147 (PMC5467134; doi:10.1089/cmb.2016.0147)

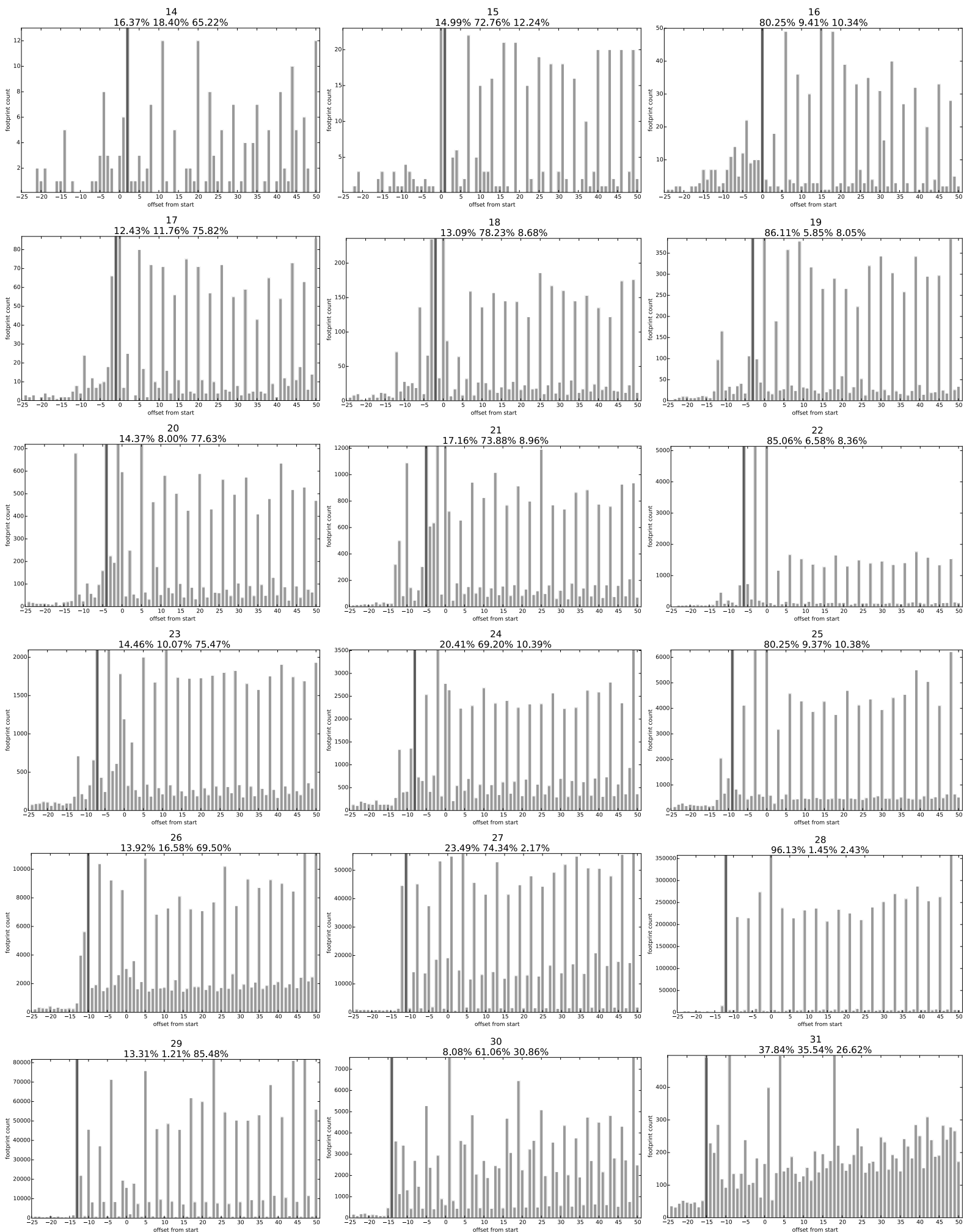

Figure S1: Metagenome profiles on reads with different lengths near the start codon.

Supplement: Supplemental data [file Supp_Fig1.pdf]
